# Identification of diagnostic biomarkers for fibromyalgia using gene expression analysis and machine learning

**DOI:** 10.3389/fgene.2025.1535541

**Published:** 2025-04-17

**Authors:** Fuyu Zhao, Jianan Zhao, Yang Li, Chenyang Song, Yaxin Cheng, Yunshen Li, Shiya Wu, Bingheng He, Juan Jiao, Cen Chang

**Affiliations:** ^1^ Department of Rheumatology, Guanghua Hospital Affiliated to Shanghai University of Traditional Chinese Medicine, Shanghai, China; ^2^ Guanghua Clinical Medical College, Shanghai University of Traditional Chinese Medicine, Shanghai, China; ^3^ Institute of Arthritis Research in Integrative Medicine, Shanghai Academy of Traditional Chinese Medicine, Shanghai, China; ^4^ Rheumatology Department, Guang’anmen Hospital, China Academy of Chinese Medical Sciences, Beijing, China; ^5^ Tongren Hospital Shanghai Jiao Tong University School Of Medicine, Shanghai, China

**Keywords:** fibromyalgia, diagnostic markers, DYRK3, RGS17, ArhGEF37

## Abstract

**Objective:**

Fibromyalgia (FM) is a complex autoimmune disorder characterized by widespread pain and fatigue, with significant diagnostic challenges due to the absence of specific biomarkers. This study aims to identify and validate potential genetic markers for FM to facilitate earlier diagnosis and intervention.

**Methods:**

We analyzed gene expression data from the Gene Expression Omnibus (GEO) to identify differentially expressed genes (DEGs) associated with FM. Comprehensive enrichment analyses, including Gene Ontology (GO), the Kyoto Encyclopedia of Genes and Genomes (KEGG), and Reactome pathways, were performed to elucidate the biological functions and disease associations of the candidate genes. We used the eXtreme Gradient Boosting (XGBoost) algorithm to develop a diagnostic model, which was validated using independent datasets.

**Results:**

Three genes, namely, dual-specificity tyrosine phosphorylation-regulated kinase 3 *(DYRK3)*, regulator of G protein signaling 17 *(RGS17),* and Rho guanine nucleotide exchange factor 37 *(ARHGEF37)*, were identified as key biomarkers for FM. These genes are implicated in critical processes such as ion homeostasis, cell signaling, and neurobiological functions, which are perturbed in FM. The diagnostic model demonstrated robust performance, with an area under the curve (AUC) of 0.8338 in the training set and 0.8178 in the validation set, indicating its potential utility in clinical settings.

**Conclusion:**

The study successfully identifies three diagnostic biomarkers for FM, supported by both bioinformatics analysis and machine learning models. These findings could significantly improve diagnostic accuracy for FM, leading to better patient management and treatment outcomes.

## 1 Introduction

Fibromyalgia (FM) is a pain syndrome characterized by widespread musculoskeletal pain (Tanwar et al., 2019), affecting millions of people worldwide and significantly impacting the quality of life. Research has found a prevalence of 2%–4% in women, which is higher than that in men (Staud, 2011), with other common symptoms, including depression, anxiety, stiffness, fatigue, sleep disturbances, and cognitive impairments (Stensson et al., 2020). Despite various studies in animals and humans revealing abnormalities in FM metabolism, biochemistry, genetics, and immune regulation, its exact causes remain unclear (Jurado-Priego et al., 2024a). The current hypothesis suggests that FM originates from interactions between the autonomic central nervous system (CNS), the hypothalamic–pituitary–adrenal axis, and the immune system (Clauw et al., 2011; Arnold, 2010; DeLeo and Yezierski, 2001; Buskila, 2001). FM is identified as a central sensitization syndrome. Central sensitization refers to a mechanism that enhances neuronal signals in the CNS, leading to heightened pain perception. In FM patients, the hallmark of central sensitization is the increased release of neurotransmitters. Among these, substance P and glutamate activate N-methyl-D-aspartic acid (NMDA) receptors that convey pain signals. Substance P, released from specific sensory nerve endings, interacts with neurokinin-1 (NK-1) receptors, and this interaction triggers the release via NMDA receptors in the spinal dorsal horn, reducing the synaptic threshold of spinal neurons (Jurado-Priego et al., 2024b). Diagnosing FM also presents several challenges. Although the diagnostic criteria released by the American College of Rheumatology (ACR) in 2021 are widely used in clinical practice, the lack of specific diagnostic biomarkers means that routine clinical laboratory diagnostics do not show objective abnormalities. Therefore, diagnosis usually occurs after other diseases are excluded based on complex clinical presentations. Due to diagnostic delays, the absence of timely treatment leads to disease progression and poor prognosis in most FM patients, causing significant distress and pressure for individuals and their families.

In the last 10 years, there have been unprecedented advances in technologies for identifying the roles of genetics and epigenetics in the development of FM, enhancing our understanding of its pathogenesis. Research indicates a significant genetic component to FM, with family studies showing that first-degree relatives face an eight times higher risk of developing FM (Dadabhoy et al., 2008). Nevertheless, it has been noted that the emergence and progression of FM may be due to the interplay of genetic, epigenetic, and environmental influences, without any single gene directly causing the condition. Recently, bioinformatics analyses and deoxyribonucleic acid (DNA) microarray gene expression profiles have become extensively applied in the genomic research of various diseases, aiding in the identification of differentially expressed genes (DEGs) and functional pathways integral to disease progression. This research leverages public data from the Gene Expression Omnibus (GEO) database to filter and examine specific gene expression microarray datasets to pinpoint DEGs relevant to FM. By employing Gene Ontology (GO) and the Kyoto Encyclopedia of Genes and Genomes (KEGG) for functional analysis, the study explores their impact on biological functions and signal pathways. Additionally, using immune infiltration analysis helps provide a more detailed view of the immunological state of FM patients, aiming to further clarify the pathological mechanisms of FM and offering insights into potential biomarkers and therapeutic targets for the disease.

## 2 Materials and methods

### 2.1 Data download and preprocessing

**FIGURE 1:**
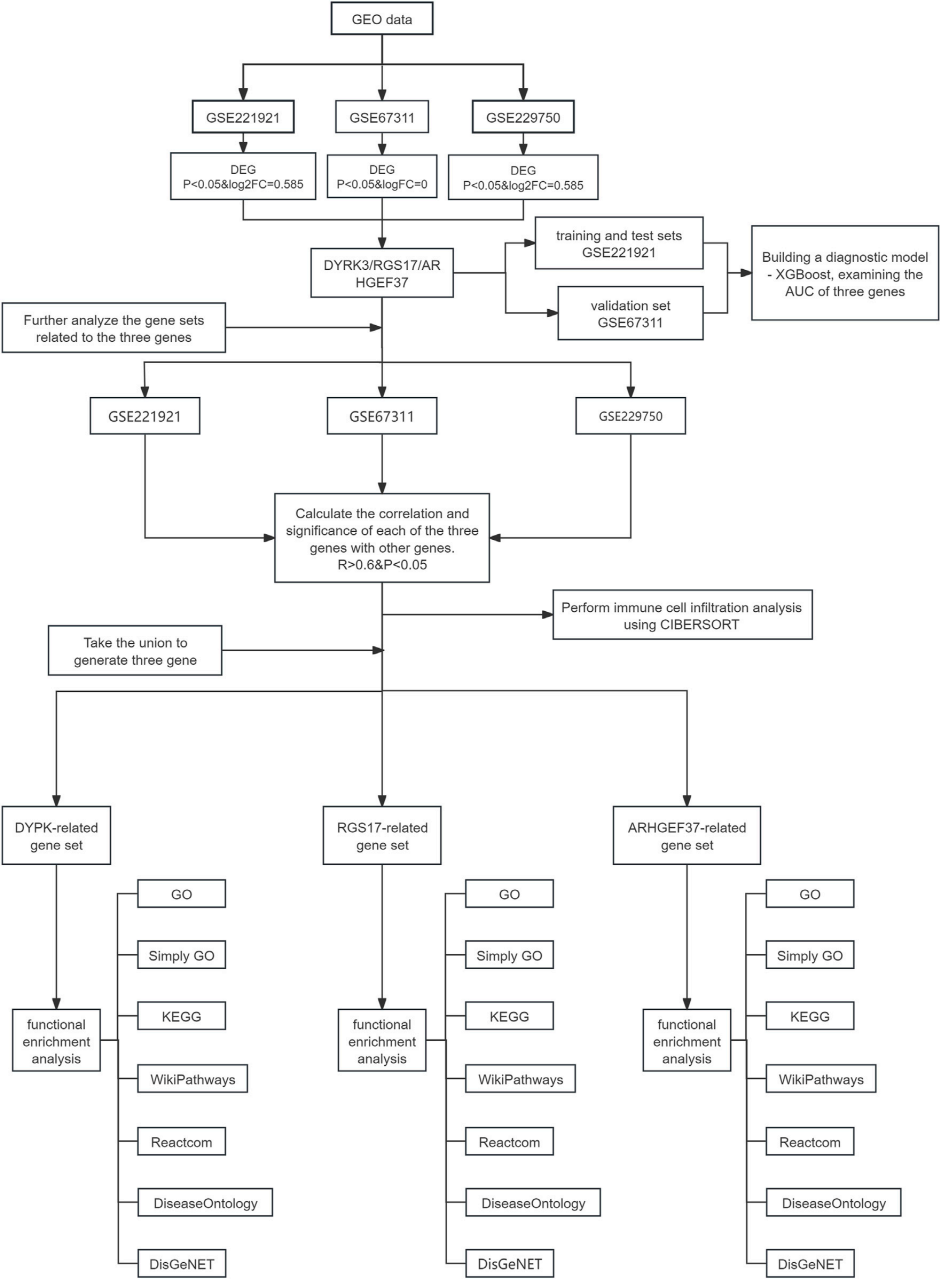
Data analysis process. This flowchart illustrates the analysis process of data obtained from the Gene Expression Omnibus database (GSE221921, GSE67311, and GSE229750), focusing on the three genes, namely, *DYRK3, RGS17*, and *ARHGEF37*. Initially, relevant datasets are extracted from the GEO database based on differentially expressed gene (DEG) analysis, and the intersection of their respective DEGs is considered to obtain the three target genes. Further analysis is conducted on gene sets related to the three genes by calculating the correlation and significance between these genes and others (R > 0.6 and P < 0.05), leading to the generation of a unified gene set comprising the three genes. A diagnostic model is built using the GSE221921 dataset with XGBoost, and this model is validated with the GSE67311 dataset to assess the AUC of the three genes. Additionally, immune cell infiltration analysis is performed using the CIBERSORT tool on each dataset to explore characteristics of the immune microenvironment. Finally, functional enrichment analyses are conducted for the DYRK3-, RGS17-, and ARHGEF37-related gene sets, using databases.

The GEO database (http://www.ncbi.nlm.nih.gov/geo) is an open-source platform created by the National Center for Biotechnology Information (NCBI) for retrieving gene expression data. This study used three public gene expression datasets, namely, GSE221921, GSE67311, and GSE229750, sourced from the GEO database. Specifically, GSE221921 comprised 189 gene expression profiles from 96 FM patients and 93 healthy controls in PBMC; GSE67311 included 140 profiles from 70 FM patients and 70 healthy controls in peripheral blood samples; and GSE229750 contained 10 profiles from five FM patients and five healthy controls in peripheral venous blood neutrophils. Differential expression analysis was independently performed for each dataset. The criteria for differential expression in GSE221921 and GSE229750 were set at a P-value <0.05 and an absolute FC ≥ 1.5; for GSE67311, the criteria were a P-value <0.05 and an absolute FC ≥ 1. The analysis identified a set of intersecting differentially expressed genes across the datasets. The specific process is shown in Figure 1.

### 2.2 Diagnostic potential analysis

Differential expression analysis was performed on GSE221921, GSE67311, and GSE229750 to obtain DEGs. The intersecting DEGs among the three datasets were identified and selected as the feature genes. Using the dataset GSE221921 as the training set and GSE67311 as the validation set, we built the model and tuned parameters on the training set, while the validation set was used to evaluate the model’s performance. The eXtreme Gradient Boosting (XGBoost) algorithm was used to construct a diagnostic model for FM. Initially, feature selection was performed on the training set, inputting the filtered differentially expressed genes into the model, and model parameters were optimized through cross-validation. The area under the receiver operating characteristic (ROC) curve (AUC) of the model was calculated using the validation set to assess the diagnostic capability of the model. In addition, the variable feature importance image was plotted.

### 2.3 Analysis of the correlation of key genes

To further validate the functional relevance of key genes, we conducted a correlation analysis on three datasets. The Spearman correlation coefficient was used to analyze the expression correlation and significance between the three intersecting genes and other genes in each dataset. The screening criteria were correlation coefficient |R| >0.6 and p-value <0.05. Gene sets related to dual-specificity tyrosine phosphorylation-regulated kinase 3 *(DYRK3),* regulator of G protein signaling 17 *(RGS17),* and Rho guanine nucleotide exchange factor 37 *(ARHGEF37)* were extracted from each dataset and combined, resulting in the final key gene sets associated with *DYRK3*, *RGS17*, and *ARHGEF37*, respectively.

### 2.4 Analysis of immune infiltration

In order to delve into the differences in immune cell composition between FM patients and healthy control groups, we performed multiple immune infiltration analyses on immune cell expression data derived from three datasets, including GSE221291. The cell-type identification by estimating relative subsets of RNA transcript (CIBERSORT) algorithm was used to estimate the relative abundance of different immune cell subgroups based on ribonucleic acid (RNA) sequencing data.

### 2.5 Analysis of functional enrichment

To investigate the potential functions of key genes in FM, we conducted various functional enrichment analyses on the extracted key gene sets. GO enrichment analysis was performed using the ClusterProfiler package to explore the enrichment of key genes in biological processes (BPs), cellular components (CCs), and molecular functions (MFs). KEGG pathway enrichment analysis was conducted using the ClusterProfiler package to explore the signaling pathways associated with key genes. Enrichment analyses using WikiPathways, Reactome, Disease Ontology, and the Disease Gene Network (DisGeNET) were carried out to delve deeper into the potential functions and related diseases of key genes in FM.

### 2.6 Statistical analysis

This study used R (version 4.3.0) for statistical analysis. Data processing and differential analysis were performed using stringr (1.5.1) (Wickham, 2023), data.table (1.16.0) (Barrett et al., 2024), GEOquery (2.72.0) (Davis and Meltzer, 2007), and edgeR (4.2.1) (Chen Y. et al., 2024). Principal component analysis (PCA) and plotting were conducted using ggsci (3.2.0) (Xiao, 2024), FactoMineR (2.11) (Le et al., 2008), factoextra (1.0.7) (Kassambara and Mundt, 2020), and corrplot (0.94) (Taiyun et al., 2024). ROC analysis was performed using shapviz (0.9.5) (Mayer, 2024), XGBoost (Chen T. et al., 2024), ROCit (Khan and Brandenburger, 2024), tibble (Müller and Wickham, 2023), caret (Kuhn, 2008), and pROC (Robin et al., 2011). Immune infiltration analysis was conducted using IOBR (0.99.9) (Zeng et al., 2021), reshape (0.8.9) (Wickham, 2007), ggplot2 (3.5.1) (Wickham, 2016), and tidyverse (2.0.0) (Wickham et al., 2019). Functional enrichment analysis was performed using clusterProfiler (4.12.6) (Xu et al., 2024; Wu et al., 2021; Yu et al., 2012), org.Hs.eg.db (3.19.1) (Carlson, 2024), and GSEABase (1.66.0) (Morgan et al., 2024).

## 3 Results

### 3.1 Differential genes for FM

**FIGURE 2:**
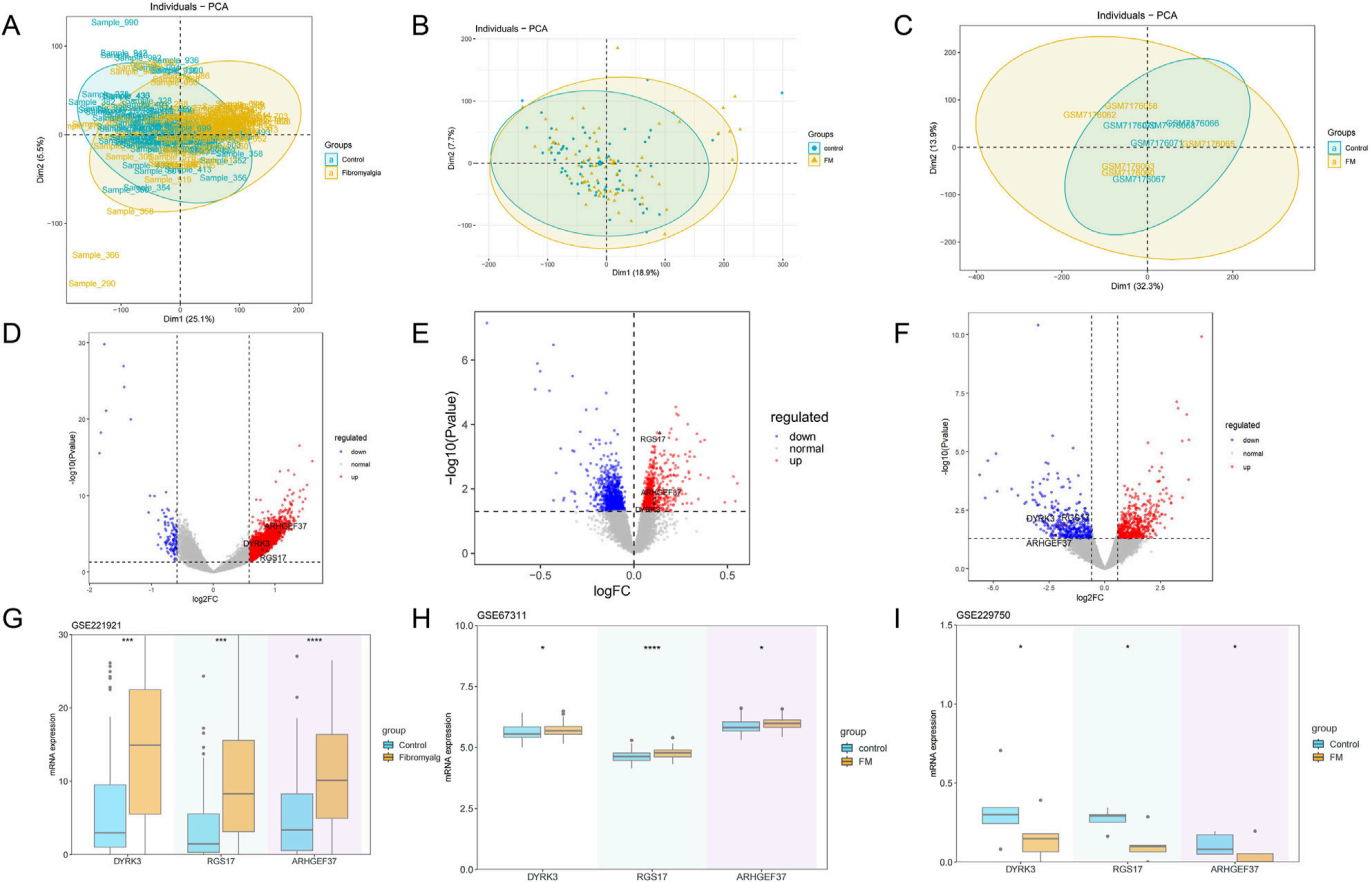
Differential gene and principal component analysis. **(A–C)** Principal component analysis of the fibromyalgia and control groups, from left to right: GSE221921, GSE67311, and GSE229750. **(D–F)** Gene expression profiles of three datasets (volcano plots), from left to right: GSE221921, GSE67311, and GSE229750. **(G–I)** Differences in gene expression between the two groups in three datasets (box plots), from left to right: GSE221921, GSE67311, and GSE229750.

PCA revealed a distinct separation in the principal component space between the control and FM groups (Figures 2A–C). Volcano plots displayed significant expression changes in hundreds of genes, with 863 genes upregulated and 867 downregulated in the GSE67311 dataset; 3,100 upregulated and 114 downregulated in the GSE221921 dataset; and 529 upregulated and 396 downregulated in the GSE229750 dataset (Figures 2D–F). The intersection of differentially expressed genes from the three datasets yielded three intersecting genes, namely, *DYRK3*, *RGS17*, and *ARHGEF37*. The genes *RGS17*, *ARHGEF37*, and *DYRK3* exhibited significant trends of upregulation or downregulation. Further box plot analyses in various GEO datasets confirmed significant differences in these genes between the two groups, underscoring their potential biological roles in FM (*P*<0.05) (Figures 2G–I). The observations offer significant insights into the molecular mechanisms underlying FM.

### 3.2 The expression of the genes *RGS17*, *ARHGEF37*, and *DYRK3* can be used as diagnostic biomarkers for FM

**FIGURE 3:**
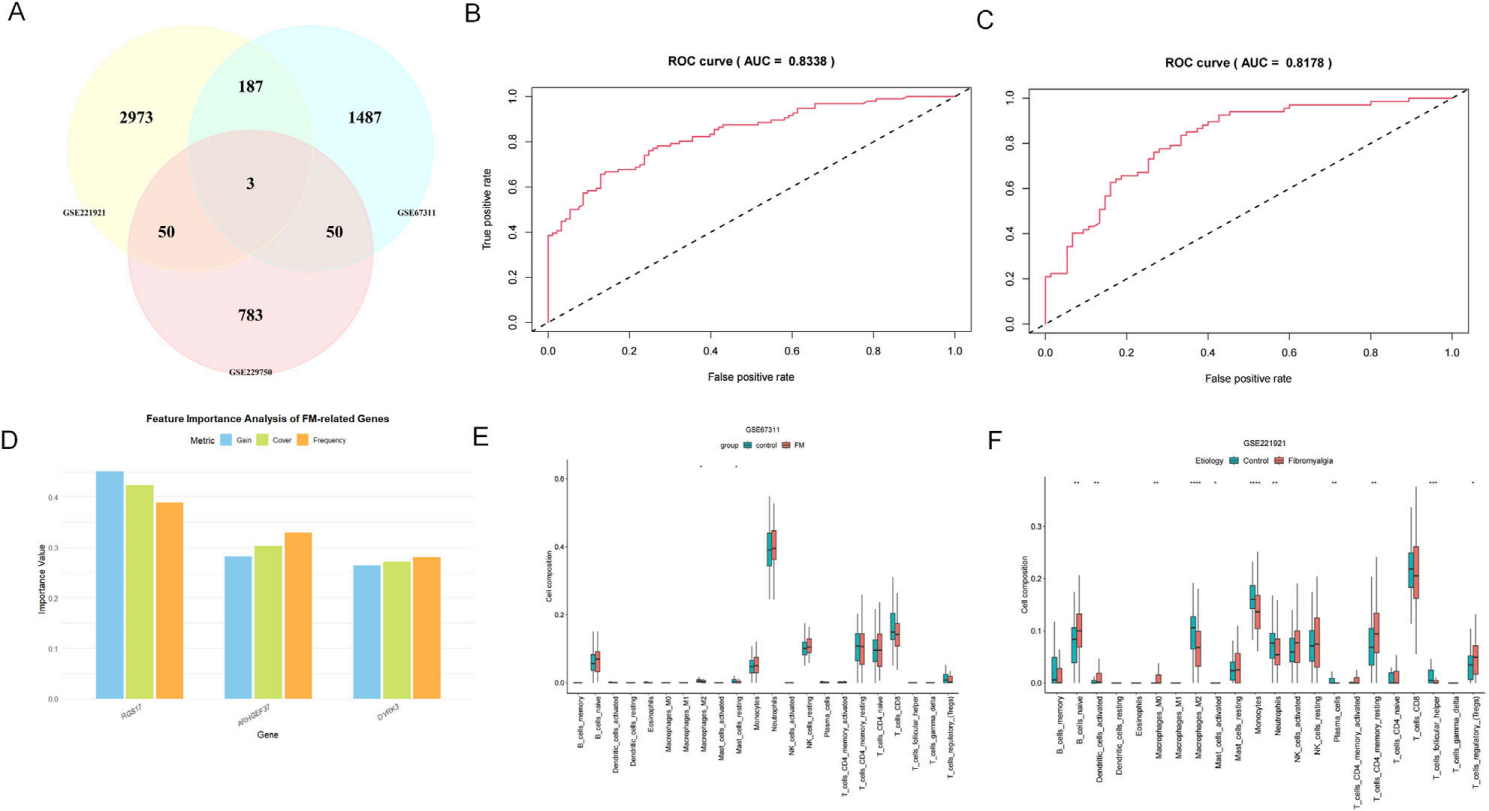
XGBoost and immune infiltration analysis. **(A)** Relevant datasets are extracted from the GEO database based on DEG analysis, and the intersection of their respective DEGs is considered to obtain the three target genes, namely, DYRK3, RGS17, and ARHGEF37. **(B, C)** ROC curves for the training and validation sets; **(D)** variable feature importance gain, the information gain brought by the gene when splitting nodes in the model (key metric); cover, the sample coverage when the gene is used for splitting; frequency, the number of times the gene appears in the trees. **(E, F)** Results of immune infiltration analysis for three gene sets (box plots).

We selected the three intersecting genes as feature genes and used them as input for the XGBoost model (Figure 3A). During model construction, hyperparameter tuning was performed using grid search. The GSE221921 dataset was used as the training set, while GSE67311 served as the validation set to develop an FM diagnostic model based on the XGBoost algorithm. The model’s diagnostic capability was evaluated by calculating the area under the ROC curve (AUC = 0.8338) using the training set (Figures 3B,C), while the AUC of 0.8178 for the validation set demonstrated the model’s strong diagnostic performance. Among the three genes, *RGS17* contributed the most to the model, indicating that its expression changes play a dominant role in FM classification decisions. *DYRK3* and *ARHGEF37* provided complementary information, potentially enhancing model performance through interactive effects. Future experimental validation should prioritize *RGS17* to assess its potential as a biomarker (Figure 3D).

### 3.3 Various differences in immune cell populations present in FM

The GSE229750 dataset shows a lower abundance of most immune cell types, with only plasma cells exhibiting differences between the two groups (*P* < 0.05). The immune infiltration results from the GSE67311 dataset reveal significant differences in the expression of inactive mast cells and M2 macrophages, with the healthy control group showing higher levels than the FM group (*P* < 0.05). In the GSE221291 dataset, there are numerous significant differences spanning various cell types, particularly evident in M2 macrophages, monocytes, and helper T cells (P < 0.05). (Figures 3E,F).

### 3.4 Functional enrichment analysis

**FIGURE 4:**
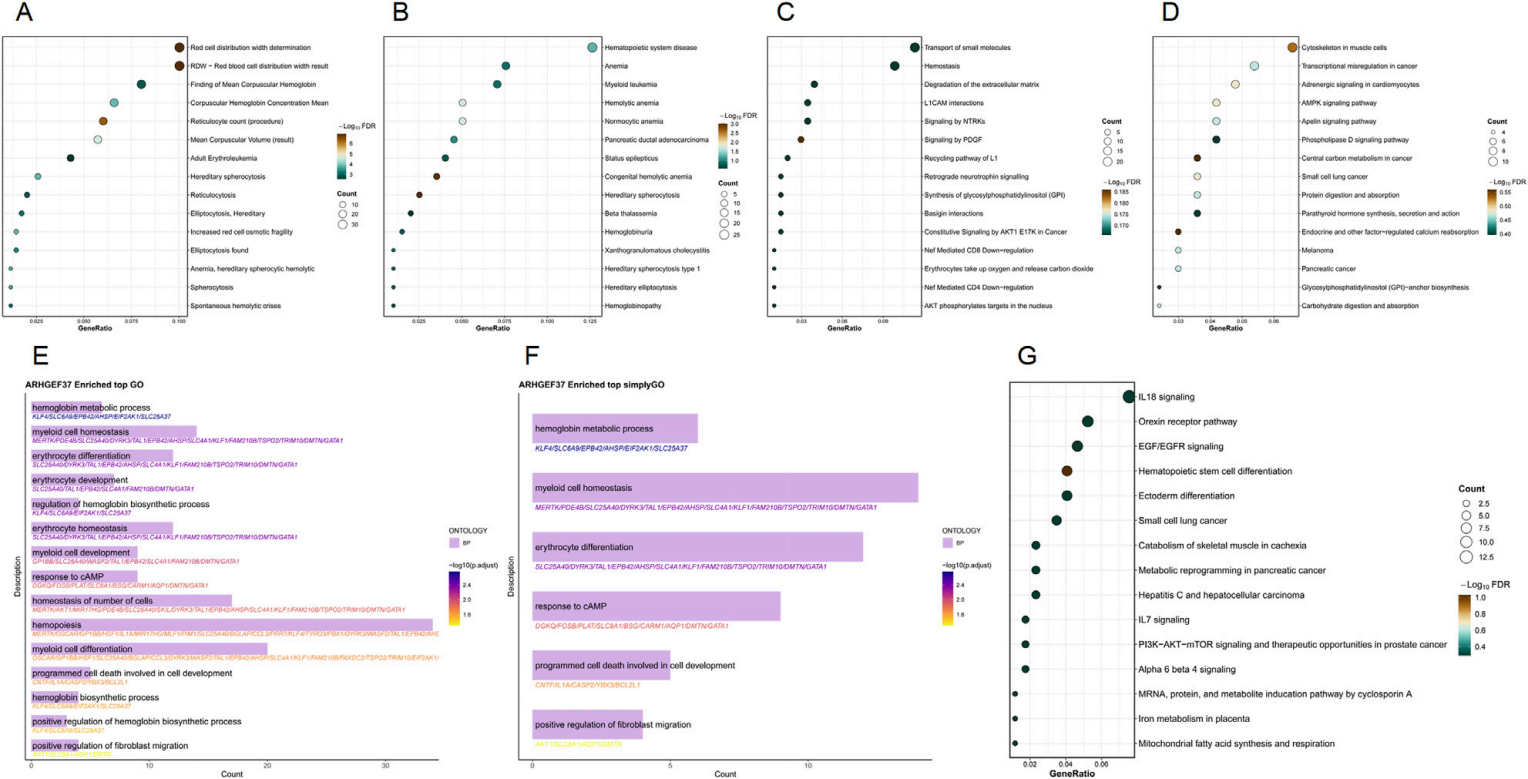
Functional enrichment analysis of the *ARHGEF37* gene set. **(A)** DisGeNET enrichment results; **(B)** Disease Ontology enrichment results; **(C)** Reactome enrichment results; **(D)** KEGG enrichment results; **(E)** GO enrichment results; **(F)** SimpleGO enrichment results; **(G)** WikiPathways enrichment results.

*ARHGEF37* is a multifunctional gene involved in key biological processes such as hematopoiesis, intracellular material transport, cell signaling, and disease progression. WikiPathways analysis links this gene to multiple signaling pathways, especially those related to inflammation, immune responses, and cancer. SimpleGO and GO analyses indicate significant enrichment in the hemoglobin metabolic process, myeloid cell homeostasis, and erythrocyte differentiation. Reactome analysis shows a higher proportion of genes involved in hemostasis and extracellular matrix degradation, along with a notable enrichment of the pathway “protein kinase B (AKT) phosphorylates targets in the nucleus” pathway. KEGG pathway analysis highlights the involvement of cytoskeletal components in muscle cell-related pathways. Disease Ontology points to its association with hematopoietic system disease, anemia, and myeloid leukemia. DisGeNET analysis reveals significant gene proportions in red cell distribution width determination and reticulocyte count and identifies hereditary disorders like hereditary spherocytosis and hereditary elliptocytosis as well-characterized conditions associated with this gene (Figure 4).

**FIGURE 5:**
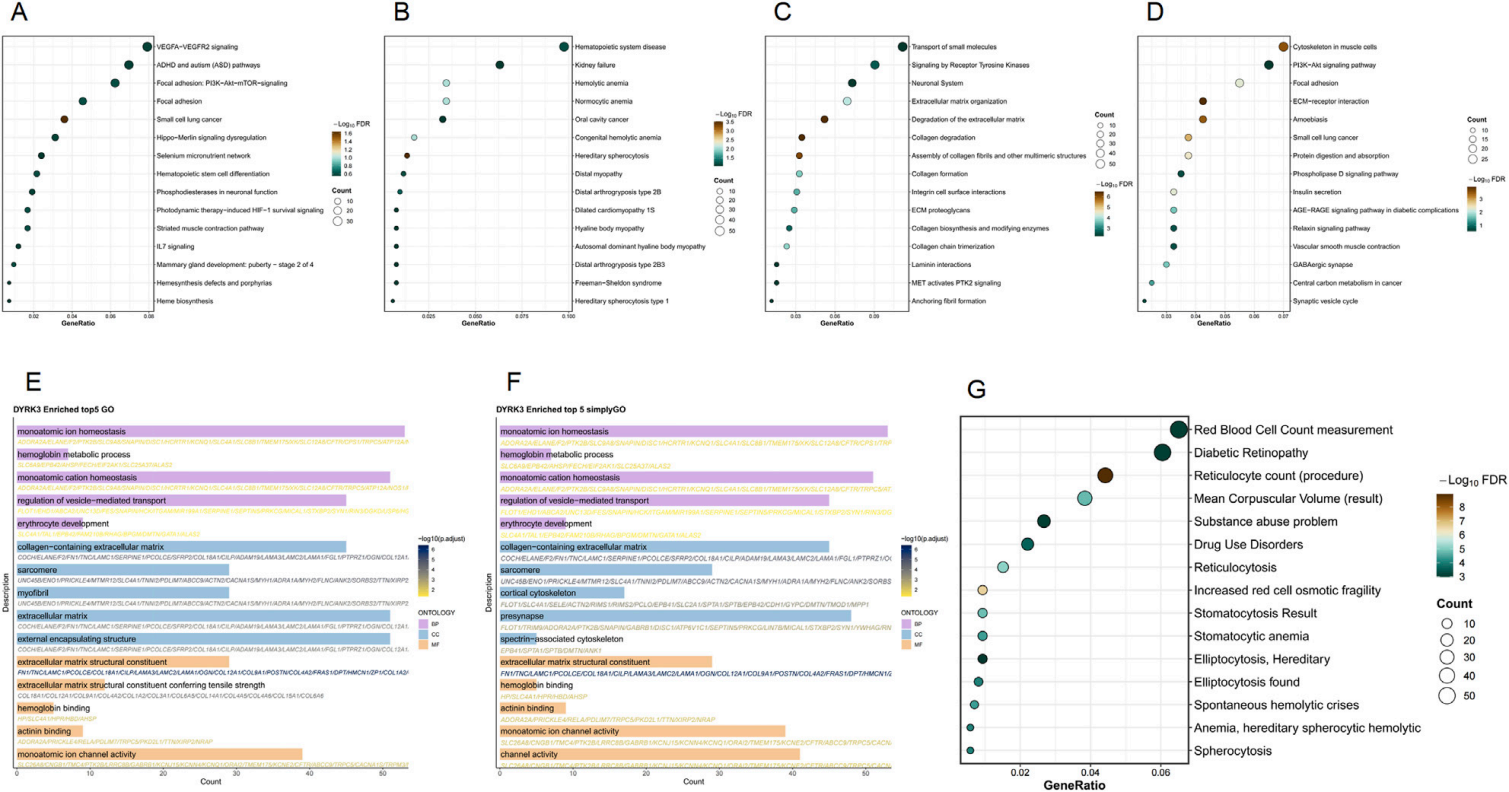
Functional enrichment analysis of the *DYRK3* gene set. **(A)** WikiPathways enrichment results; **(B)** Disease Ontology enrichment results; **(C)** Reactome enrichment results; **(D)** KEGG enrichment results; **(E)** GO enrichment results; **(F)** SimpleGO enrichment results; **(G)** DisGeNET enrichment results.

The *DYRK3* gene significantly impacts cell biology and disease progression. In WikiPathways analysis, it prominently features in vascular endothelial growth factor A (VEGFA)–vascular endothelial growth factor receptor 2 (VEGFR2) signaling, phosphoinositide 3-kinase (PI3K)–AKT–mammalian target of rapamycin (mTOR) signaling in focal adhesion, and small-cell lung cancer pathways. SimpleGO analysis highlights substantial enrichment in the hemoglobin metabolic process, monoatomic ion homeostasis, and erythrocyte differentiation. GO analysis indicates notable involvement in monoatomic ion homeostasis, channel activity, extracellular matrix-related functions, including collagen content—all showing high gene counts and statistical significance. Reactome analysis underscores its role in signaling by receptor tyrosine kinases and extracellular matrix organization, supported by high gene proportions and significance. KEGG pathway analysis identifies extensive involvement in cytoskeletal organization within muscle cell-related pathways. Disease Ontology points to strong associations with hematopoietic system diseases, including hemolytic anemia and hereditary spherocytosis. In DisGeNET, red cell distribution width determination, diabetic retinopathy, and reticulocyte count (procedure) show high gene proportions and pronounced color enrichment (Figure 5).

**FIGURE 6:**
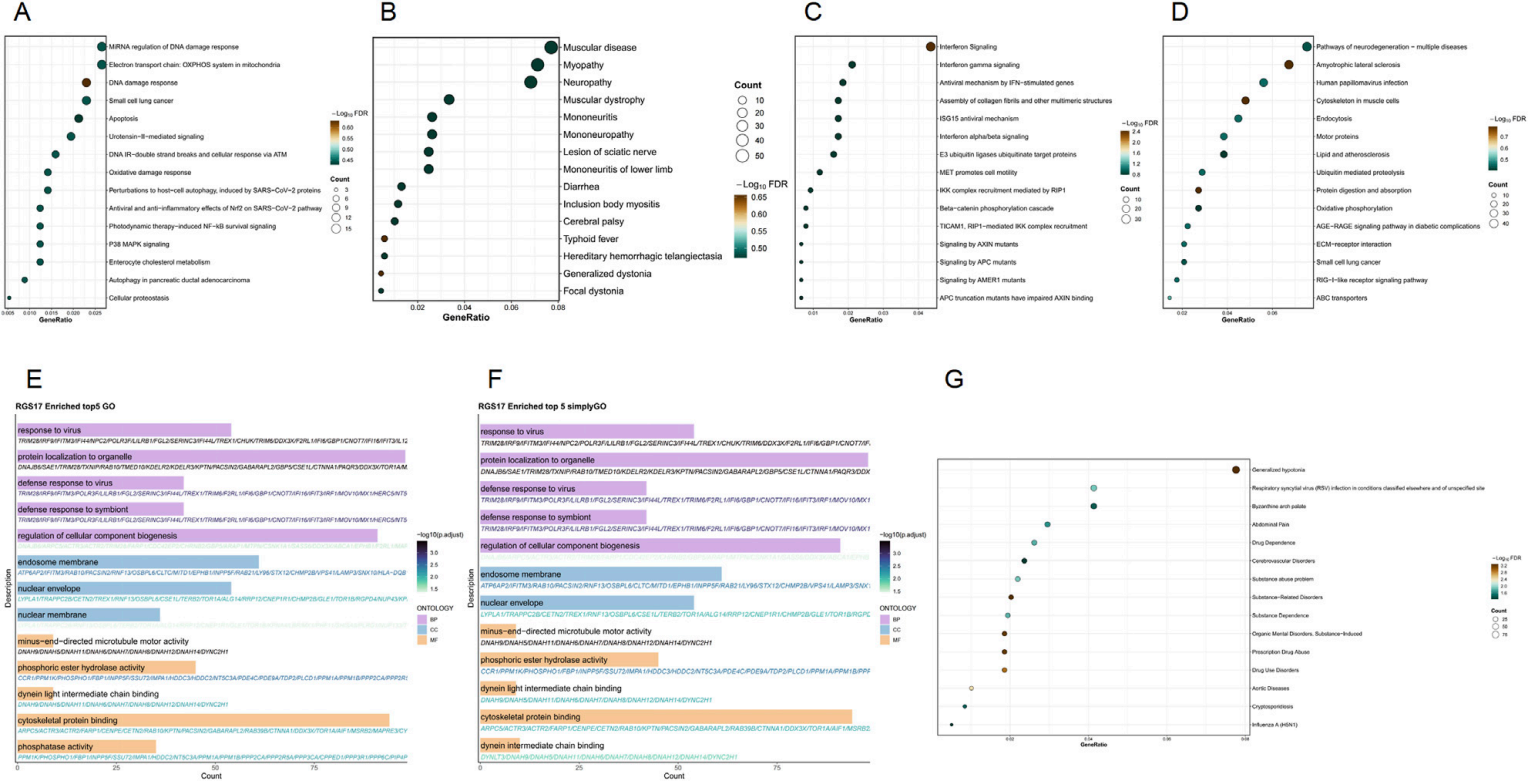
Functional enrichment analysis of the *RGS17* gene set. **(A)** WikiPathways enrichment results; **(B)** Disease Ontology enrichment results; **(C)** Reactome enrichment results; **(D)** KEGG enrichment results; **(E)** GO enrichment results; **(F)** SimpleGO enrichment results; **(G)** DisGeNET enrichment results.

The enrichment analysis of the *RGS17* gene reveals its potential role in various biological processes. WikiPathways analysis highlights its role in miRNA regulation of the DNA damage response and the DNA damage response itself, showing relatively high gene proportions and statistical significance. SimpleGO and GO analysis indicate notable involvement in viral responses, defense against viruses, the endosomal membrane, and the nuclear envelope, with substantial gene counts and significance. In Reactome, interferon signaling and interferon gamma signaling are marked by relatively high gene proportions and significance. KEGG pathways such as pathways of neurodegeneration–multiple diseases and amyotrophic lateral sclerosis also show high gene proportions and significance. Disease Ontology identifies significant associations with muscular diseases, myopathies, and neurological conditions such as neuropathy and mononeuropathy. DisGeNET indicates notable gene proportions in generalized hypotonia and respiratory syncytial virus (RSV) infection, as well as in drug-related disorders like drug dependence and substance abuse (Figure 6).

## 4 Discussion

FM is a complex and heterogeneous condition. The variety of symptoms and the absence of early diagnostic indicators mean that FM is often diagnosed only after excluding other conditions, resulting in delayed treatment. The diagnosis of FM remains a controversial topic, with continuously evolving standards and assessment methods reflecting the complexity of the condition. Throughout the literature, it is clear that there is ongoing debate and exploration regarding various aspects of FM, including its etiology, diagnosis, and management (Jurado-Priego et al., 2024a).

In recent years, bioinformatics and the GEO datasets have played an increasingly important role in the diagnosis and treatment of diseases. Bioinformatics provides powerful tools for understanding the molecular mechanisms of diseases, identifying new biomarkers, and developing targeted treatment strategies by integrating and analyzing large amounts of data. The GEO dataset, as a public repository of gene expression data, contains various experimental data from around the world, which also accelerates scientific discoveries. A search revealed that there are only three datasets related to FM, reflecting the complexity of diagnosing and treating the condition. Therefore, further in-depth exploration of FM using bioinformatics and the GEO datasets is beneficial for advancing research in its diagnosis and treatment.

From the initial screening of differentially expressed genes across three datasets, the intersecting genes *DYRK3*, *RGS17*, and *ARHGEF37* were identified, which may act as biomarkers for diagnosing FM. Additionally, to further validate the functional relevance of these three genes, we conducted correlation analyses on the three datasets and obtained the final key gene sets related to *DYRK3*, *RGS17*, and *ARHGEF37*, followed by functional enrichment analysis of these key gene sets.

Wash-free infiltration analysis indicated significant differences in the abundance of various cell types compared to those in the control group, particularly in monocytes, mast cells, and M2 macrophages. These differences suggest a significant divergence in immune cell composition between FM patients and healthy individuals, possibly reflecting abnormal immune regulation in the disease state. Studies have shown that inflammation, particularly neuroinflammation and autoimmunity, plays a role in the pathogenesis of FM (Theoharides et al., 2019). Mast cells are a part of the immune system, primarily involved in allergic reactions, and can release inflammatory mediators such as histamine and prostaglandins. An animal study showed that mast cells mediate pain and fatigue behaviors, representing potential therapeutic targets for treating FM syndrome (Brum et al., 2024). M2 macrophages are expressed significantly less in the FM group than in the control group; these cells are usually associated with anti-inflammatory responses and tissue repair and can inhibit inflammation by releasing anti-inflammatory cytokines such as interleukin 10 (IL-10) and transforming growth factor beta (TGF-β). There is ample evidence that anti-inflammatory cytokines have analgesic effects in animal models (Sommer et al., 2018), suggesting that the dysfunction of M2 macrophages may be associated with FM. Additionally, monocytes are activated in the inflammatory environment within the body, producing various cytokines, including anti-inflammatory and pro-inflammatory factors, which may affect the symptoms of FM patients. The relationship between monocytes and the development of FM requires further research. It is undeniable that the immune infiltration analysis of the three datasets did not yield consistent results, which may be related to different disease states, time points, or intervention factors. Additionally, the limited number of datasets may also have influenced the results to some extent. Despite observing different immune infiltration characteristics across the datasets, these findings still provide valuable insights.

*DYRK3* is part of the dual-specificity tyrosine phosphorylation-regulated kinase family, and its cellular functions are not yet fully understood. Studies have found that *DYRK3* phosphorylates SNAP-associated protein (SNAPIN), which positively regulates dynamin-mediated mitochondrial retrograde transport and soluble N-ethylmaleimide-sensitive factor attachment protein receptor (SNARE) complex-mediated exocytosis of neuronal synaptic vesicles, indicating that *DYRK3* affects cell vitality and provides a novel neuroprotective mechanism (Kim et al., 2021). FM is a chronic pain syndrome characterized by neuroinflammation and impaired oxidative balance in the central nervous system. Studies have confirmed that mitochondrial dysfunction plays a key role in the pathogenesis of FM (Marino et al., 2024). Functional enrichment analysis reveals that the expression and regulation of the *DYRK3* gene play crucial roles in intracellular signal transmission, cellular structure maintenance, and the development of specific diseases. In terms of cellular signaling, *DYRK3* is highly enriched in several critical cellular pathways, particularly those involved in erythrocyte development and hemoglobin metabolism. Regarding cell structure and tissue integrity, *DYRK3* shows enrichment in the extracellular matrix and structures associated with the cell membrane, involving the regulation of the cytoskeleton and the function of the nuclear membrane. In terms of disease association, *DYRK3* is closely related to various blood diseases, such as hereditary spherocytosis, and some neurological conditions. These conditions often involve abnormalities in erythrocytes or degenerative changes in neuronal cells, suggesting that *DYRK3* might serve as a biomarker for diagnosing FM.

*RGS17*, a compelling drug target, belongs to the RGS Z (RZ) subfamily and is predominantly expressed in the central nervous system (Hayes et al., 2021). Research has found that five RGS members are abundantly expressed in the brain, with *RGS17* notably expressed in the striatum and significantly enriched in the cerebellum. This research reveals the roles of individual RGS members in various human functions and supports the involvement of several RGS members in regulating central nervous system functions through G-protein-coupled receptor (GPCR)-mediated signal transduction (Larminie et al., 2004). Functional enrichment analysis has shown its potential roles in various biological processes, with changes in *RGS17* gene expression associated with multiple disease states under pathological conditions, particularly in diseases affecting the respiratory, nervous, and cardiovascular systems and in conditions related to drug dependency. Currently, further research is lacking on the relationship between FM and *RGS17*. Martins et al. (2017) indicated that physical exercise can activate multiple intracellular pathways via GPCRs, mitigating FM pain symptoms by inhibiting elevated phosphorylation of protein kinase A (PKA). This could be an important direction for future research into the relationship between *RGS17* and the pathogenesis of FM.

*ARHGEF37* is a guanine nucleotide exchange factor (GEF) that regulates the Rho family of small guanosine triphosphatases (GTPases). Functional enrichment analysis has highlighted its potential roles across various biological processes, disease states, and cellular functions. In terms of biological processes and signaling pathways, this gene is significantly enriched in red blood cell differentiation, hematopoiesis, and hemoglobin biosynthesis, suggesting that it may play a crucial role in the development of blood diseases. In cellular functions, it may play a regulatory role in organelle function and cell signaling. In terms of disease association, *ARHGEF37* is linked with various disease states, including muscular diseases, neurodegenerative disorders, and certain infectious diseases. Current research on *ARHGEF37* is limited, mainly focusing on its role in promoting adhesion and transendothelial migration of tumor cells in hepatocellular carcinoma and enhancing the infiltration and metastatic capabilities of hepatocellular carcinoma (HCC) cells (Zhang et al., 2022); there is a lack of research on other diseases, such as FM. Enrichment analysis shows that this gene is involved in several key biological processes, such as hematopoiesis, intracellular material transport, cell signaling, and disease progression, which require further exploration.

Our data indicate that the XGBoost-predictive model constructed using *DYRK3, RGS17*, and *ARHGEF37* performs well in distinguishing between FM and healthy individuals. During the modeling process, we used a grid search strategy for hyperparameter tuning. We carefully selected a set of key hyperparameters, including learning rate (eta), tree depth (max_depth), subsample rate (subsample), colsample_bytree, and gamma, which play a critical role in controlling the model’s complexity and generalization ability. Specifically, the learning rate determines the contribution of each individual tree to the final prediction. A higher learning rate may lead to faster convergence but increases the risk of overfitting, while a lower learning rate generally ensures more gradual learning but requires more rounds for convergence. The maximum depth of the trees influences the model’s ability to capture complex relationships between features; deeper trees can capture more intricate patterns but are also more prone to overfitting. The subsample rate controls the proportion of training data used for each tree, with lower values introducing randomness and helping prevent overfitting. Gamma, as a regularization parameter, penalizes splits with minimal gain, thereby reducing the complexity of the trees and improving generalization. The minimum child weight (min_child_weight) parameter ensures that splits only occur when there is a sufficient amount of data in the child node, thereby preventing overly complex splits. Through grid search and cross-validation, we fine-tuned these hyperparameters to achieve strong performance on the training set while ensuring the model generalized well on the test set. It is important to note that slight changes in these hyperparameters could significantly alter the model’s performance, and we aimed to select settings that strike a balance between accuracy and overfitting prevention. The size of the training dataset is another crucial factor affecting the performance of the XGBoost model. In our study, we used a relatively small dataset consisting of three genes (*ARHGEF37, DYRK3*, and *RGS17*), and the model performed well despite the limited size. However, small datasets pose challenges such as overfitting, particularly when the number of features is large compared to the number of samples. In our case, the number of features (genes) was much smaller than the number of samples, which mitigated the risk of overfitting. Nevertheless, we used cross-validation to assess the model’s generalizability and obtain more robust performance estimates. The size of the training data affects how well the model can capture underlying patterns in the data. Larger datasets generally provide more diverse information, which can help the model generalize better. Although our dataset was small, we were able to achieve good performance through regularization techniques and cross-validation. Increasing the dataset size in future studies could potentially improve model robustness and prediction accuracy. In summary, the performance of the XGBoost model in this study was influenced by the careful tuning of hyperparameters such as the learning rate, tree depth, and subsample rate. Moreover, the small size of the training dataset posed challenges such as the potential for overfitting, but the model still performed well due to the use of regularization techniques and cross-validation. We acknowledge that both hyperparameters and training data size are important factors in the model’s performance, and future studies could explore the impact of increasing the dataset size and further optimizing hyperparameters to refine model performance.

In summary, this study identified three intersecting genes, namely, *DYRK3*, *RGS17*, and *ARHGEF37*, as potential diagnostic biomarkers for FM by screening and analyzing differentially expressed genes from the FM GEO database. By conducting correlation analysis to obtain the corresponding gene sets and performing functional enrichment analysis, the study further explored the potential relevance of the three intersecting genes in the pathogenesis of FM and their potential as diagnostic markers. However, due to the lack of in-depth research on the pathogenesis of FM and the functions of these three genes, further exploration is required. Additionally, as this study lacks validation with clinical data, future research will focus on verifying these findings using larger-scale clinical datasets to further assess their diagnostic value and potential clinical applications. This will be a crucial direction for future studies.

## Data Availability

The original contributions presented in the study are included in the article/supplementary material; further inquiries can be directed to the corresponding authors.

## Glossary

FM: Fibromyalgia
GEO: Gene Expression Omnibus
GO: Gene Ontology
DYRK3: Dual-specificity tyrosine phosphorylation-regulated kinase 3
RGS17: Regulator of G protein signaling 17
ARHGEF37: Rho guanine nucleotide exchange factor 37
XGBoost: eXtreme Gradient Boosting
NMDA: N-methyl-D-aspartic acid receptor
NK-1: Neurokinin-1
DNA: Deoxyribonucleic acid
CNS: Central nervous system
KEGG: Kyoto Encyclopedia of Genes and Genomes
NCBI: National Center for Biotechnology Information
FC: Fold change
ROC: Receiver operating characteristic
AUC: Area under the curve
CIBERSORT: Cell-type identification by estimating relative subsets of RNA transcripts
RNA: Ribonucleic acid
BPs: Biological processes
CCs: Cellular components
MFs: Molecular functions
DisGeNET: Disease Gene Network
PCA: Principal component analysis
AKT: Protein kinase B
VEGFA: Vascular endothelial growth factor A
VEGFR2: Vascular endothelial growth factor receptor 2
mTOR: Mammalian target of rapamycin
RSV: Respiratory syncytial virus
IL-10: Interleukin 10
TGF-β: Transforming growth factor Beta
SNAPIN: SNAP-associated protein
SNARE: Soluble N-ethylmaleimide-sensitive factor attachment protein receptor
GPCRs: G-protein-coupled receptors
RZ: Regulator of G-protein signaling Z
PKA: Protein kinase A
GEF: Guanine nucleotide exchange factor
GTPases: Guanosine triphosphatases
HCC: Hepatocellular carcinoma.
